# Deciphering metabolic disease mechanisms for natural medicine discovery via graph autoencoders

**DOI:** 10.3389/fphar.2025.1594186

**Published:** 2025-04-23

**Authors:** Qingquan Liao, Wei Zhao, Zhan Wang, Lei Xu, Kun Yang, Xinxin Liu, Lichao Zhang

**Affiliations:** ^1^ Department of Information Technology, Hunan Police Academy, Changsha, China; ^2^ School of Electronic and Communication Engineering, Shenzhen Polytechnic University, Shenzhen, China; ^3^ School of Data Science and Artificial Intelligence, Wenzhou University of Technology, Wenzhou, China; ^4^ School of Intelligent Manufacturing and Equipment, Shenzhen Institute of Information Technology, Shenzhen, China

**Keywords:** metabolic diseases, natural medicines, drug discovery, graph autoencoder, metabolite-disease associations

## Abstract

Metabolic diseases, such as diabetes, pose significant risks to human health due to their complex pathogenic mechanisms, complicating the use of combination drug therapies. Natural medicines, which contain multiple bioactive components and exhibit fewer side effects, offer promising therapeutic potential. Metabolite imbalances are often closely associated with the pathogenesis of metabolic diseases. Therefore, metabolite detection not only aids in disease diagnosis but also provides insights into how natural medicines regulate metabolism, thereby supporting the development of preventive and therapeutic strategies. Deep learning has shown remarkable efficacy and precision across multiple domains, particularly in drug discovery applications. Building on this, We developed an innovative framework combining graph autoencoders (GAEs) with non-negative matrix factorization (NMF) to investigate metabolic disease pathogenesis via metabolite-disease association analysis. First, we applied NMF to extract discriminative features from established metabolite-disease associations. These features were subsequently integrated with known relationships and processed through a GAE to identify potential disease mechanisms. Comprehensive evaluations demonstrate our method’s superior performance, while case studies validate its capability to reveal pathological mechanisms in metabolic disorders including diabetes. This approach may facilitate the development of natural medicine-based interventions. Our data and code are available at: https://github.com/Lqingquan/natural-medicine-discovery.

## Introduction

In recent years, the incidence and mortality rates of metabolic diseases, such as diabetes, have risen sharply (Neel and Sargis, 2011). These diseases affect a broad population, and their complex pathogenic mechanisms present significant treatment challenges. Synthetic small-molecule drugs typically target only one or a few pathways, whereas the treatment of metabolic diseases often requires combination therapies, increasing the risk of side effects and complications (Makhoba et al., 2020). Natural medicines, an integral part of traditional medical knowledge, have accumulated extensive experience in disease prevention, diagnosis, and treatment. They generally contain multiple bioactive compounds that act on diverse molecular targets while exhibiting relatively fewer side effects. Therefore, leveraging natural medicines for the treatment of metabolic diseases, including diabetes, represents a promising research direction (Ansari et al., 2023). With the rapid advancement of artificial intelligence (AI), significant breakthroughs have been achieved in information processing and medical applications (Xu et al., 2021). AI has demonstrated substantial potential in analyzing complex biomedical data, particularly in drug discovery and disease diagnosis, creating new opportunities for developing natural medicine-based therapies for metabolic diseases. In medical research, understanding the relationship between metabolite levels and disease pathogenesis is essential. For example, blood glucose and glycosylated hemoglobin measurements effectively assess diabetes progression (Welsh et al., 2016). Additionally, natural compounds such as piperine and their metabolites exhibit potential therapeutic effects on cardiovascular and hepatic diseases (Azam et al., 2022). Therefore, precise metabolite detection not only facilitates disease diagnosis but also advances research on how natural medicines regulate metabolic processes.

Conventional methods for assessing metabolite levels and investigating metabolic disease pathogenesis rely heavily on clinical observations and biochemical assays. While these methods yield robust data, their high resource and labor requirements present significant constraints. To address these limitations, computational approaches have emerged as powerful tools for elucidating disease mechanisms. For instance, Hu et al. utilized known metabolite-disease interactions from the HMDB database (Wishart et al., 2022) to construct a metabolite interaction network, applying a random walk algorithm to identify novel associations (Hu et al., 2018). Lei et al. introduced a computational model integrating disease semantic information and Gaussian interaction profile (GIP) similarity, leveraging the KATZ algorithm to predict unknown metabolite-disease connections (Lei and Zhang, 2019). Expanding on this work, Lei et al. further incorporated disease functional similarity, along with GIP and metabolite functional similarities, employing a bipartite graph recommendation algorithm for improved prediction accuracy (Lei and Zhang, 2020). Zhao et al. fused multiple metabolite and disease similarity measures to construct a similarity network, extracting features using graph convolutional networks and employing deep neural networks to predict novel metabolite-disease relationships (Zhao et al., 2021). Zhang et al. applied three distinct feature extraction techniques combined with principal component analysis (PCA) to refine metabolite-disease pair representations, classifying them using the LightGBM algorithm (Zhang et al., 2021). Unlike other approaches, Tie et al. integrated information entropy similarity of diseases and metabolites during feature extraction, utilizing a random forest algorithm to infer potential associations (Tie et al., 2021). Sun et al. constructed a heterogeneous network, extracting features through graph neural networks and decoding them to reconstruct a metabolite-disease interaction network (Sun et al., 2022). Gao et al. employed multiple feature extraction techniques to separately process metabolite and disease features, concatenating them to form metabolite-disease pair representations, which were subsequently classified using a multilayer perceptron (MLP) (Gao et al., 2023). These computational approaches have significantly advanced research on the pathogenesis of metabolic diseases, facilitating the identification of novel disease mechanisms and potential therapeutic targets.

Natural medicines are derived from natural sources, such as plants, and have contributed to the development of numerous modern drugs, including aspirin, artemisinin, and paclitaxel (Gurib-Fakim, 2006). Their discovery typically involves extracting active ingredients from natural resources and identifying potential drug candidates through bioactivity screening (Lahlou, 2007). The advancement of computational methods in drug discovery has further facilitated the development of natural medicines (Zhou et al., 2024a). For example, Zhou et al. employed a subgraph-based approach to extract local topological features of drugs and proteins, integrating an energy-constrained diffusion mechanism to capture global interactions, thereby identifying novel drug-protein interactions (Zhou et al., 2024b). Additionally, Zhou et al. incorporated autoencoder technology based on a similar framework to accurately predict microbial responses to drugs (Zhou et al., 2024c). Wei et al. developed a drug-target interaction prediction method combining ensemble learning and deep learning techniques, optimizing performance through clustering and fine-tuning base learner parameters (Wei et al., 2024a). They also explored potential food-drug relationships using self-supervised learning (Wei et al., 2024b). Furthermore, Wei et al. introduced a novel framework for drug repositioning that integrates multi-source prompting and large language model technology, highlighting the critical role of large language models in this field (Wei et al., 2024c). Since drug discovery encompasses both synthetic and natural drugs, these advanced computational techniques can also accelerate the identification and development of novel natural medicines.

Despite significant advancements in metabolite-disease association (MDA) prediction, several challenges remain. First, existing methods primarily construct complex similarity networks to extract features, which may limit model generalization. Second, due to inherent limitations in data collection, datasets inevitably contain noise. To address these issues, we propose a novel method which integrates NMF with GAE technology to improve the accuracy of MDA predictions. Initially, we employ NMF to extract the initial representations of metabolites and diseases from known MDAs, eliminating the need for complex similarity networks. Next, we apply a Bernoulli sampling strategy to randomly mask a subset of known MDAs, reducing the influence of noisy data. Finally, we utilize a GAE, leveraging an encoder-decoder framework to reconstruct the metabolite-disease network. Our contributions can be summarized as follows:

(1) We successfully identified potential MDAs by integrating NMF with GAE technology, achieving superior predictive performance.
(2) We employed NMF to extract initial representations from known MDAs, reducing dependence on complex similarity networks and enhancing model generalization.
(3) We implemented a Bernoulli-based masking strategy to mitigate the impact of noise in the dataset, further refining metabolite and disease representations through an in-depth analysis of metabolite and disease neighbor densities.
(4) We conducted comprehensive case studies on diabetes, liver diseases, and gastrointestinal diseases, analyzing their associated metabolites. Additionally, we performed multiple experiments to thoroughly assess the effectiveness of the models.

## Materials and methods

This study proposes a novel MDA prediction model based on NMF and GAE technology. Compared to traditional prediction models, the proposed model introduces several key innovations. First, it utilizes NMF to extract initial representations of metabolites and diseases from known MDAs, eliminating the need for multiple similarity networks. Second, it employs a Bernoulli sampling strategy to randomly mask a subset of known associations, mitigating the impact of noisy data. Third, it leverages GAE technology within an encoder-decoder framework to achieve precise reconstruction of the metabolite-disease network.

### Data preparation

Metabolite and disease data were extracted from the Human Metabolome Database (HMDB), with missing values removed during preprocessing (Hu et al., 2018). The final curated dataset comprises 4,536 MDAs involving 2,262 metabolites and 216 diseases. These associations include common metabolic diseases such as uremia, leukemia, and hepatitis. During the experiment, we represented MDAs as an adjacency matrix ***A*** of dimensions ***U*** × ***V***, where ***U*** is the number of metabolites and ***V*** is the number of diseases.

### Model framework

**FIGURE 1:**
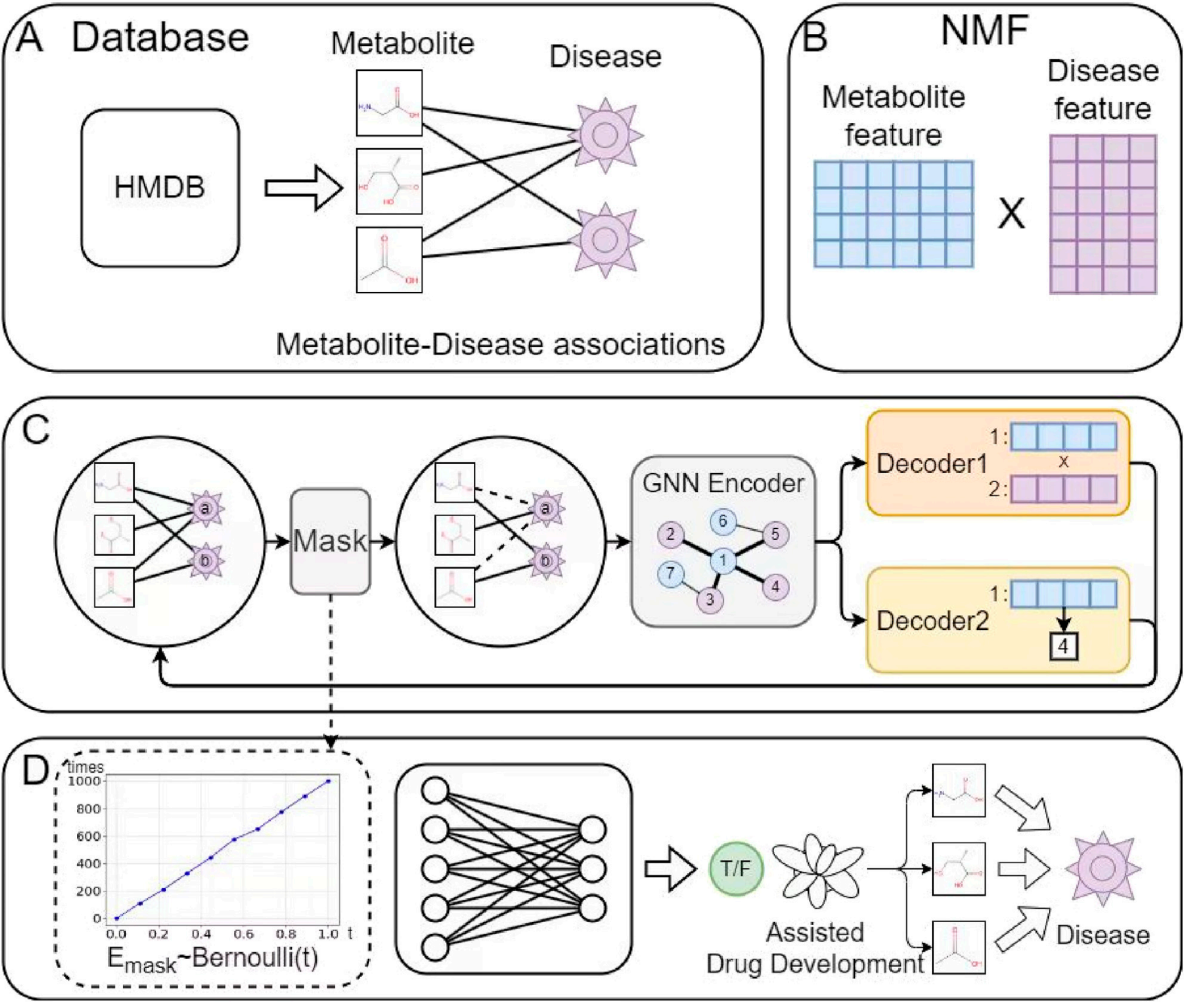
The proposed model architecture comprises four main components: **(A)** Data preprocessing: cleaning raw data by removing noise and handling missing values; **(B)** Feature extraction: employing NMF to obtain initial metabolite and disease representations; **(C)** GAE: reconstructing the metabolite-disease network via an encoder-decoder architecture while learning node representations; **(D)** MDA prediction: identifying potential associations using the learned metabolite-disease representations.

Figure 1 illustrates the architecture of the proposed model, which comprises four main components: (A) Data preparation, (B) Initial feature extraction for metabolites and diseases, (C) GAE, and (D) MDA prediction. In module (A), observed MDA data were collected from the HMDB database. Based on this data, module (B) applies an alternating iterative method using Tucker decomposition and least squares to derive initial feature matrices for metabolites and diseases. Next, module (C) employs Bernoulli-based sampling to mask parts of the metabolite-disease graph before inputting it into the graph neural network (GNN) encoder. Decoder1 performs vector inner product operations on metabolite-disease pairs to obtain their final representations. Simultaneously, Decoder2 supervises the reconstruction process by constraining the neighborhood density of metabolite and disease nodes to enhance biological realism. Finally, module (D) predicts MDA scores and assigns labels accordingly.

### Feature extraction

In the past decade, NMF methods have achieved significant success across various fields, including recommendation systems (Marcuzzo et al., 2022). The core concept involves approximating the original user-item matrix by deriving low-dimensional vectors for users and items, which allows for the accurate prediction of unknown associations. These methods typically utilize parallel computing techniques to capture low-dimensional feature vectors, enabling high-speed and precise predictions. This advantage extends to feature extraction in biological networks. For example, Ding et al. applied NMF to the miRNA-disease matrix to extract features of miRNAs and diseases (Ding et al., 2021). Building on this approach, our study plans to apply NMF to the metabolite-disease network to efficiently and accurately extract preliminary features of metabolites and diseases. Compared to traditional biological network feature extraction methods, the primary advantage of NMF is that it eliminates the need for constructing various similarity networks, thereby enhancing the model’s generalization ability.

Given the MDA matrix $A_{U\times V}$ , our goal is to derive low-dimensional vector matrices $M_{U\times K}$ and $D_{K\times V}$ for metabolites and diseases, respectively, such that their product closely approximates $A_{U\times V}$ . In this decomposition, each column vector in A is expressed as a weighted sum of the corresponding column vectors in $M_{U\times K}$ , with the weights determined by the respective column vectors in $D_{K\times V}$ . Additionally, the K must satisfy the constraint K<U,V and $K<UV/\left(U+V\right)$ . Based on this formulation, A is decomposed into M and D . This study employs Tikhonov regularization as the optimization objective, as shown in Equation 1:

$$\min_{M\geq 0,D\geq 0}{\left\|A⊙\left(A-MD\right)\right\|}_{F}^{2}+\mu_{1}{\left\|M\right\|}_{F}^{2}+\mu_{2}{\left\|D\right\|}_{F}^{2}$$ (1)

where ${\left\|\cdot \right\|}_{F}$ denotes the Frobenius norm of a matrix, $\mu_{1}$ and $\mu_{2}$ represent the regularization coefficients for the low-dimensional representations of metabolites and diseases, respectively. In this study, both $\mu_{1}$ and $\mu_{2}$ are set to 0.01 by default, and the K is fixed at 90. Directly solving for matrices M and D is computationally challenging. A widely used approach to simplify this problem is the alternating least squares (ALS) method, which iteratively updates M and D . Based on this, the Lagrangian optimization objective is formulated as Equation 2:

$$L\left(M,D\right)={\left\|W⊙\left(A-MD\right)\right\|}_{F}^{2}+\mu_{1}Tr\left(MM^{T}\right)+\mu_{2}Tr\left(DD^{T}\right)+Tr\left(\gamma M^{T}\right)+Tr\left(\pi D^{T}\right)$$ (2)

where $\gamma =\left(\gamma_{ik}\right)$ and $\pi =\left(\pi_{kj}\right)$ are Lagrange multipliers, $Tr\left(\cdot \right)$ represents the trace of a matrix, and ⊙ represents the Hadamard product operation. We take the partial derivative as Equation 3:

$$\frac{\partial L}{\partial M}=-2\left(W⊙\left(A-\text{MD}\right)\left(D^{T}\right)\right)+2\mu_{1}M+\gamma =-2\left(\left(W⊙A\right)D^{T}\right)+2\left(W⊙\left(\text{MD}\right)D^{T}\right)+2\mu_{1}M+\gamma$$ (3)

$$\frac{\partial L}{\partial D}=-2\left(M^{T}\left(W⊙\left(A-MD\right)\right)\right)+2\mu_{2}D+=-2\left(M^{T}\left(W⊙A\right)\right)+2\left(M^{T}\left(W⊙\left(MD\right)\right)\right)+2\mu_{2}D+\pi$$ (4)

Let $\gamma_{ik}M_{ik}=0$ , $\gamma_{ik}M_{ik}=0$ . According to the Tucker decomposition rule (Kim and Choi, 2007), the update formula for the low-dimensional vector matrices of metabolites and diseases is given by Equations 5, 6, respectively:

$$M_{ik}^{t}\leftarrow M_{ik}^{t-1}\frac{{\left(\left(W⊙A\right)D^{T}\right)}_{ik}}{{\left(W⊙\left(MD\right)D^{T}+\mu_{1}M\right)}_{ik}}$$ (5)

$$D_{kj}^{t}\leftarrow D_{kj}^{t-1}\frac{{\left(M^{T}\left(W⊙A\right)\right)}_{kj}}{{\left(M^{T}\left(W⊙\left(MD\right)\right)+\mu_{2}D\right)}_{kj}}$$ (6)

where $M_{ik}^{t}$ represents the value of the element in the ***i***-th row and ***k***-th column of the metabolite low-dimensional matrix at the ***t***-th iteration. By specifying the number of iterations, the low-dimensional vector matrices $M_{U\times K}$ and $D_{K\times V}$ for metabolites and diseases, as well as the MDA matrix $A_{U\times V}$ , are obtained and subsequently used as input for the GAE.

### Graph autoencoder

Graph autoencoders stem from the graph encoder-decoder architecture, which effectively maps complex node and edge relationships into a low-dimensional space. Due to this capability, they have been widely applied in recommendation systems and biological networks (Malla and Banka, 2023). In this study, we employ GAE technology to reconstruct potential metabolite-disease networks. First, based on previous research, we utilize Bernoulli sampling to randomly mask a portion of observed MDAs, mitigating the impact of noisy data. Next, the GNN encoder extracts representations of metabolites and diseases within the masked metabolite-disease network. Subsequently, a MLP decodes MDAs, while a degree decoder analyzes the neighbor density of metabolites and diseases.

#### Masking based on Bernoulli distribution

Due to limitations in experimental observation, environmental factors, and measurement technology, the collected metabolite-disease network data may contain errors. Noise data comprises inaccurate, incomplete, or irrelevant observations introduced during data collection. Such data deviate from ground truth and may represent errors or redundancies. In metabolite-disease association datasets, noise manifests as misclassification between MDA and non-MDA pairs. Furthermore, data collection errors for metabolites or diseases constitute another significant noise source. To address this, Hou et al. mitigated the impact of noise by masking portions of the graph’s topological structure (Hou et al., 2022). Inspired by their approach, our study employs a Bernoulli distribution-based sampling strategy to suppress noise interference in the metabolite-disease network. Specifically, in each iteration of training, a subset of MDAs is sampled according to the Bernoulli distribution as shown in Equation 7:

$$E_{mask}∼Bernoulli\left(\tau \right)$$ (7)

where τ denotes the masking rate of the metabolite-disease network, ranging from [0,1], while $E_{mask}$ represents the masked MDAs. Based on this, the masked MDAs can be derived as Equation 8:

$$E_{reserved}=E_{all}-E_{mask}$$ (8)

where $E_{reserved}$ denotes the reserved MDAs, while $E_{all}$ represents all observed MDAs. Subsequently, the decoder reconstructs the masked MDAs, thereby completing the training process. By applying this masking strategy, we aim to mitigate the adverse effects of noise in the metabolite-disease network and improve model performance. Masked MDAs remain positive samples during training, though excluded from GNN encoder message passing. Each training epoch reapplies Bernoulli distribution-based random masking to MDAs.

#### GNN encoder

This study employs the Graph Isomorphism Network (GIN) as the encoder, which primarily functions to compute node representations by aggregating neighborhood information. Other common GNNs, such as Graph Convolutional Networks (GCN) (Kipf and Welling, 2016) and Graph Attention Networks (GAT) (Veličković et al., 2017), could also serve as alternatives. For the MDA prediction task, each metabolite or disease node in the metabolite-disease graph aggregates its own information along with that from its neighboring nodes. Subsequently, a MLP is used to map the aggregated information into the latent space. The process of deriving metabolite or disease representations can be defined as Equations 9, 10, respectively:

$$H_{m,a}^{t}={MLP}^{t}\left(\left(1+\epsilon^{t}\right)\cdot H_{m,a}^{t-1}+\sum_{b\in N\left(a\right)}H_{d,b}^{t-1}\right)$$ (9)

$$H_{d,b}^{t}={MLP}^{t}\left(\left(1+\epsilon^{t}\right)\cdot H_{d,b}^{t-1}+\sum_{a\in N\left(b\right)}H_{m,a}^{t-1}\right)$$ (10)

where $H_{m,a}^{t}$ and $H_{d,b}^{t}$ represent the features of metabolite ***a*** and disease ***b*** at the ***t***-th layer of GIN, respectively. ${MLP}^{t}$ denotes the parameters of the ***t***-th layer of GIN, while $\epsilon^{t}$ represents the trainable parameters at this layer, facilitating the integration of node information with its neighborhood. $N\left(a\right)$ and $N\left(b\right)$ indicate the neighborhoods of metabolite ***a*** and disease ***b***, respectively. Given a specific layer ***t***, the final representations of metabolites and diseases are obtained and randomly fed into the decoder.

#### Decoder

This study employed two decoders: one for reconstructing MDAs and the other for imposing constraints based on the neighborhood density of metabolite or disease nodes. Both decoders utilized an MLP architecture.

In the first decoder, the representation of a metabolite-disease pair is inputted and processed through an MLP. Generally, this representation can be defined using the Hadamard product, vector inner product, vector addition, or vector concatenation. For example, when using vector concatenation, the pair $\langle a,b\rangle$ , representing metabolite ***a*** and disease ***b***, is expressed as $MLP\left(H_{m,a}^{t}|H_{d,b}^{t}\right)$ . The decoder then predicts a score for the pair $\langle a,b\rangle$ . Consequently, the loss for reconstructing the metabolite-disease network is computed using the BCE function, as shown in Equation 11:

$$L_{edge}=\sum_{l}^{L}\left(y_{l}-1\right)\log\left(1-y_{l}^{gt}\right)-y_{l}\log\left(y_{l}^{gt}\right)$$ (11)

where L represents the total number of metabolite-disease pairs, $y_{l}$ represents the predicted score for the first metabolite-disease pair, ranging from [0,1]. $y_{l}^{gt}$ represents the true label for the first metabolite-disease pair, where values of {0,1} indicate the presence or absence of an association.

The second decoder models the neighborhood of metabolite or disease nodes to constrain the reconstruction of the metabolite-disease network during training. This study employs the mean squared error (MSE) method to compute the regression loss between the predicted and true degrees of metabolite or disease nodes, as shown in Equation 12.

$$L_{degree}=\frac{1}{U+V}\sum_{s=1}^{U+V}{\left(y_{s}-p_{s}\right)}^{2}$$ (12)

where U+V represents the total number of metabolites and diseases, $y_{s}$ represents the true degree of the ***s***-th metabolite or disease, and $p_{s}$ represents the degree predicted by the model. Based on this, the model iteratively refines the training process using the degree loss $L_{degree}$ , ensuring that the predicted values align more closely with the actual data.

#### Training and inference

As outlined in the previous process, the MDA reconstruction loss, denoted as $L_{edge}$ , is computed using a graph encoder-decoder architecture. Additionally, the MSE method is employed to compute the regression loss between the predicted and actual degrees of metabolite and disease nodes. During training, a linear additive strategy integrates the losses from both decoders, as shown in Equation 13:

$${L=L}_{edge}+lL_{degree}$$ (13)

where l is a weight parameter, which balances the contributions of the two decoders.

After training for a predefined number of iterations, the reconstructed metabolite-disease network is obtained. At this stage, metabolite and disease features are extracted, and the representation of a metabolite-disease pair is derived using the vector dot product. The final score for each pair is predicted using a MLP, as shown in Equation 14:

$$y_{a,b}=MLP\left(H_{m,a}^{T}\cdot H_{d,b}\right)$$ (14)

where $H_{m,a}$ and $H_{d,b}$ represent the final representations of metabolite ***a*** and disease ***b***, respectively. The predicted score $y_{a,b}$ quantifies the model’s confidence in the association between ***a*** and ***b***.

## Results

This study evaluated the performance of the proposed model against several advanced models. These models incorporate various cutting-edge algorithms, including Random Walk with Restart (RWR) (Wishart et al., 2022) (random walk-based), PageRank (Yates and Dixon, 2015) (ranking-based), KATZ (Lei and Zhang, 2019) (information metric-based), the Ensemble Kernel Ridge Regression (EKRR) algorithm (Peng et al., 2020) (ensemble learning-based), Graph Convolutional Network Attention (GCNAT) (Sun et al., 2022) and Deep-DRM (Zhao et al., 2021) (GNN-based), as well as the MDA-AENMF algorithm (Gao et al., 2023) (GAE-based). Additionally, multiple ablation experiments were conducted to assess the contributions of key modules in the proposed model to overall performance. Model stability was further analyzed through parameter sensitivity experiments, and recommendations for parameter selection were provided. Finally, in-depth case studies on diabetes, liver diseases, and gastrointestinal diseases were performed, examining the metabolite components associated with these conditions.

### Experimental setup

To ensure a fair comparison, all models were evaluated using five-fold cross-validation. The default parameter configuration included: masking rate (0.4), weight l (0.6), encoder dimensions [64, 128], and decoder dimensions [128, 64]. We employed the Adam optimizer with a fixed learning rate of 0.001. The model was trained on the complete masked metabolite-disease graph without batch partitioning. Following previous studies (Chen et al., 2024), we primarily used Area Under the Curve (AUC), Area Under the Precision-Recall curve (AUPR), Accuracy (ACC), Precision (PRE), Sensitivity (SEN), F1-Score (F1), and Matthews Correlation Coefficient (MCC) as evaluation metrics. The AUC measures the entire two-dimensional area underneath the Receiver Operating Characteristic (ROC) curve, with its calculation defined as Equation 15:

$$\text{AUC}=\int_{0}^{1}\text{TPR}\left(\text{FPR}\right)\text{dFPR},\text{TPR}=\frac{\text{TP}}{\text{TP}+\text{FN}},\text{FPR}=\frac{\text{FP}}{\text{FP}+\text{TN}}$$ (15)

The ROC curve graphically represents the trade-off between the true positive rate (TPR) and false positive rate (FPR) across different classification thresholds, where TP (true positives) and TN (true negatives) denote correctly classified MDA and non-MDA instances, while FP (false positives) and FN (false negatives) indicate misclassified cases. The F1-score represents the harmonic mean of precision and recall, providing a balanced metric that accounts for both measures, with its calculation defined as Equation 16:

$$F1-\text{score}=\frac{2\cdot \text{Precision}\cdot \text{Recall}}{\text{Precision}+\text{Recall}}$$ (16)

where $\text{Precision}=\frac{\text{TP}}{\text{TP}+\text{FP}}$ measures the ratio of correctly predicted MDAs to all predicted MDAs, and $\text{Recall}=\frac{\text{TP}}{\text{TP}+\text{FP}}$ indicates the ratio of correctly predicted MDAs to all actual MDAs. The AUPR quantifies the area beneath the precision-recall curve, particularly valuable for evaluating models on imbalanced datasets, with its calculation defined as Equation 17:

$$\text{AUPR}=\int_{0}^{1}\text{Precision}\left(\text{Recall}\right)\text{dRecall}$$ (17)

### Performance evaluation

**FIGURE 2:**
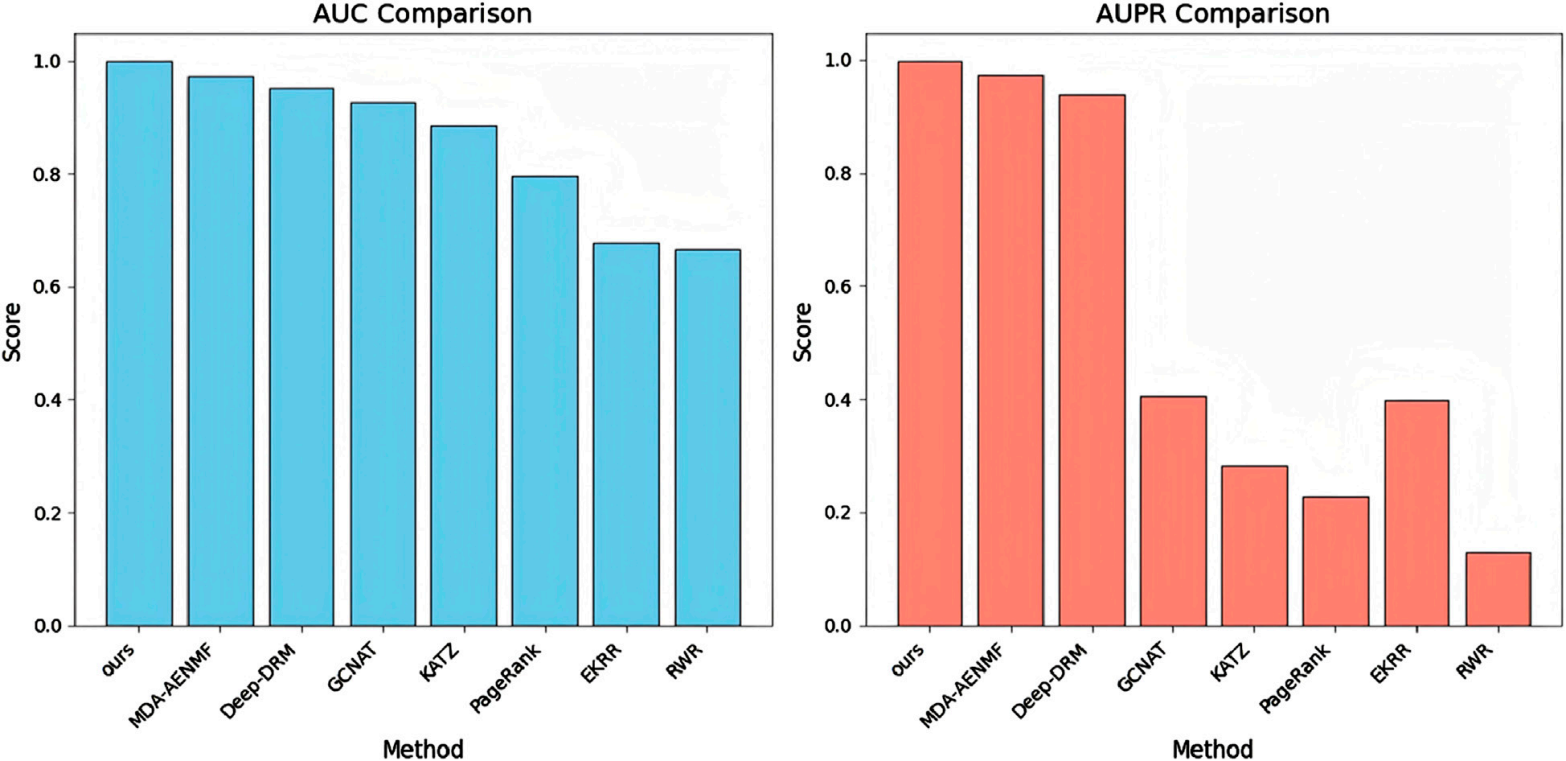
AUC and AUPR scores of the proposed model and all comparative models.

Figure 2 presents the AUC and AUPR scores of the proposed model and all comparative models. The results indicate that all models achieve higher AUC scores than AUPR scores, particularly the GCNAT, KATZ, PageRank, EKRR, and RWR algorithms. This discrepancy may be due to their negative sampling strategy, where all unobserved metabolite-disease pairs are considered negative samples. In contrast, the proposed model, MDA-AENMF, and Deep-DRM models adopt a 1:1 positive-to-negative sample ratio, leading to higher AUPR scores. In terms of AUPR performance, traditional machine learning models such as KATZ, PageRank, and RWR perform the worst. The EKRR algorithm, which incorporates ensemble learning, shows slight improvements, underscoring the advantages of ensemble learning over traditional methods. Additionally, models based on GNN, including GCNAT and Deep-DRM, as well as those using GAEs, such as MDA-AENMF and the proposed model, demonstrate superior performance. This highlights the importance of extracting topological information from the metabolite-disease network for accurate association prediction. Notably, the GAE-based MDA-AENMF and proposed models outperform the GNN-based GCNAT and Deep-DRM models, suggesting that GAEs can capture deeper structural information and enhance node representations. Among all models, the proposed model achieves the highest AUC and AUPR scores, demonstrating its effectiveness in MDA prediction.

Our analysis reveals several performance-limiting constraints in existing methods. RWR’s dependence on graph topology leads to degraded performance on sparse networks. PageRank emphasizes node centrality while neglecting metabolite-disease relationships. KATZ exhibits high parameter sensitivity. EKRR’s multi-model integration risks overfitting. GNN-based methods (GCNAT, Deep-DRM) are vulnerable to structural incompleteness and noise. MDA-AENMF processes similarity networks separately, potentially missing heterogeneous metabolite-disease interactions. While RWR, PageRank and KATZ capture network topology, their inability to extract deep features limits prediction accuracy. In contrast, GNN/GAE-based methods (GCNAT, Deep-DRM, MDA-AENMF) excel at capturing both topological features and deep dependencies, yielding superior performance.

**FIGURE 3:**
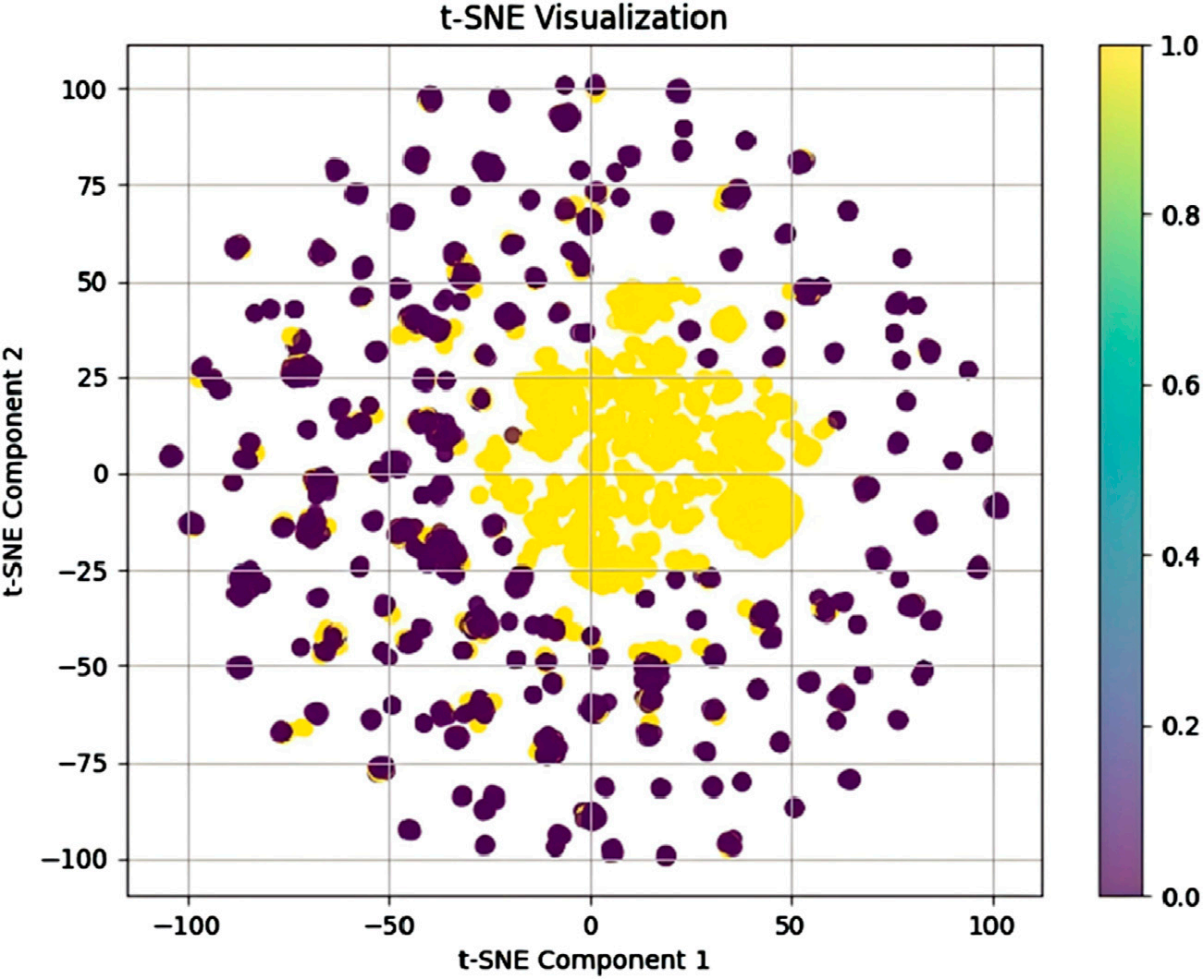
Visualization of the model-learned metabolite-disease embeddings using t-SNE dimensionality reduction.

To further evaluate the model’s performance and minimize the influence of random factors, we conducted a five-fold cross-validation experiment. Table 1 presents the results of the five-fold cross-validation for the proposed model. On average, the proposed model achieved an AUC of 0.9981, AUPR of 0.9970, ACC of 0.9912, SEN of 0.9894, PRE of 0.9928, SPE of 0.9930, F1-score of 0.9911, and MCC of 0.9825. These results further confirm the strong adaptability and reliability of the proposed model. Additionally, visualization of the model-learned metabolite-disease embeddings using t-SNE dimensionality reduction effectively demonstrates its feature extraction capability. Accordingly, we combined the model-generated metabolite and disease embeddings to construct the final metabolite-disease representations. These representations were subsequently visualized using t-SNE, as shown in Figure 3. In the visualization, yellow and purple dots denote MDA and non-MDA instances, respectively. The visualization reveals two distinct clusters: a central cluster of MDA points (yellow) and a peripheral cluster of non-MDA points (purple). This clear separation demonstrates our model’s effectiveness in extracting discriminative metabolite-disease representations, enabling accurate prediction of unknown metabolite-disease pairs.

**TABLE 1:** Results of 5-fold cross validation of proposed model.

Rounds/Metrics	AUC	AUPR	ACC	SEN	PRE	SPE	F1	MCC
1	0.9957	0.9937	0.9664	0.9471	0.9851	0.9857	0.9657	0.9334
2	0.9983	0.9966	0.9978	1.0000	0.9956	0.9956	0.9978	0.9956
3	0.9997	0.9996	0.9978	1.0000	0.9956	0.9956	0.9978	0.9956
4	0.9984	0.9979	0.9972	1.0000	0.9945	0.9945	0.9973	0.9945
5	0.9986	0.9970	0.9967	1.0000	0.9934	0.9934	0.9967	0.9934
Avg	0.9981	0.9970	0.9912	0.9894	0.9928	0.9930	0.9911	0.9825

In summary, we hypothesize that, beyond the GAE’s ability to effectively capture topological information from the metabolite-disease network, the superior performance of the proposed model may stem from several key factors. First, the proposed model utilizes NMF to extract initial features of metabolites and diseases without relying on complex similarity networks, thereby enhancing scalability. Second, it incorporates a masking strategy based on the Bernoulli distribution, which reduces the impact of noisy data and improves model robustness. Third, during training, a decoder constrained by node neighborhoods regulates the decoding process of metabolites and diseases, ensuring better alignment with real-world scenarios.

### Ablation experiments

To evaluate the contributions of key components in the proposed model, we conducted ablation experiments. The results, presented in Table 2, highlight the impact of removing individual modules on overall model performance. In the ablation study, three key components were selectively excluded: “w/o d” refers to the model without the neighborhood-based decoder constraint, “w/o m” refers to the model without the Bernoulli distribution-based masking strategy, “w/o n” refers to the model without the NMF module for feature extraction. The removal of any module led to a decline in performance metrics, particularly ACC, SEN, PRE, SPE, F1, and MCC, confirming the positive contributions of these components. Notably, the most significant performance drop was observed when the masking module was removed, indicating its critical role in mitigating the influence of noisy data and enhancing model robustness. The exclusion of the NMF module or the neighborhood-based decoder resulted in similar declines, suggesting that both modules contribute equally to overall model effectiveness. These findings reinforce the necessity of incorporating all three key components to optimize the performance of the proposed model.

**TABLE 2:** Results of ablation experiments for proposed model.

Methods/metrics	AUC	AUPR	ACC	SEN	PRE	SPE	F1	MCC
w/o d	0.9883	0.9883	0.9438	0.9184	0.9675	0.9691	0.9423	0.8887
w/o m	0.9553	0.9625	0.8881	0.8037	0.9668	0.9724	0.8778	0.7875
w/o n	0.9860	0.9835	0.9399	0.9702	0.9147	0.9096	0.9417	0.8814
Ours	0.9986	0.9970	0.9967	1.0000	0.9934	0.9934	0.9967	0.9934

### Parameter experiments

The proposed model’s architecture integrates a Bernoulli sampling-based masking module, a GAE, and a dual-decoder module. Key parameters include the types and layers of GNN encoders, the masking ratio, and the influence of the neighborhood decoder. To assess model stability and optimize parameter selection, we conducted a series of experiments evaluating the impact of these parameter variations on performance.

**FIGURE 4:**
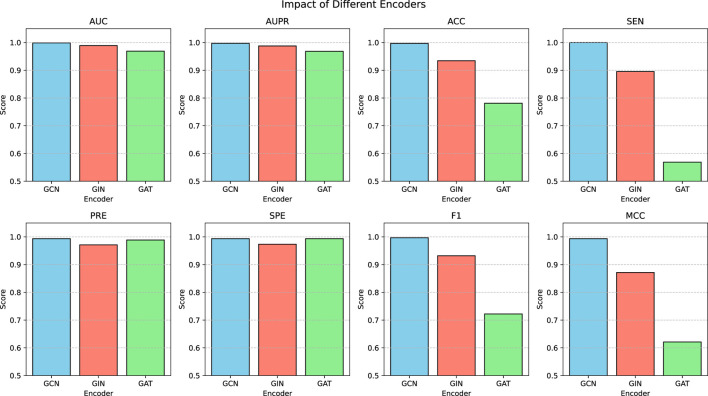
Model performance using different GNN encoders.

The proposed model is based on a graph encoder-decoder architecture, with multiple GNN models available for the graph encoder. GCN employs Laplacian matrix-based graph convolution to aggregate neighborhood information. GIN utilizes a weighted aggregation mechanism to combine node features with neighborhood information. This architecture excels in graph isomorphism detection and demonstrates superior performance in graph classification tasks. GAT leverages an attention mechanism to dynamically weight and aggregate neighborhood information, making it particularly effective for heterogeneous graph processing. To assess the stability of the model under different encoder configurations, we conducted a series of comparative experiments. Figure 4 presents the performance of the proposed model using various GNN encoders. The results indicate that the model achieved satisfactory AUC, AUPR, PRE, and SPE metrics with GCN, GIN, and GAT encoders. However, when employing GIN or GAT, the ACC, SEN, F1, and MCC metrics declined, with the GAT encoder yielding the poorest performance. This suggests that GIN and GAT may be less effective in identifying MDAs, leading to higher false-negative rates. GAT dynamically adjusts node weights based on neighborhood density, increasing the influence of densely connected nodes while reducing that of sparse nodes. Additionally, GAT’s sensitivity to noise further contributes to its suboptimal performance. Meanwhile, GIN requires large volumes of high-quality training data to mitigate overfitting. In contrast, the proposed model performed optimally when using the basic GCN encoder, likely due to its simple structure, which adapts more effectively to different architectures. Thus, for similar datasets, GCN is recommended as the preferred encoder.

**FIGURE 5:**
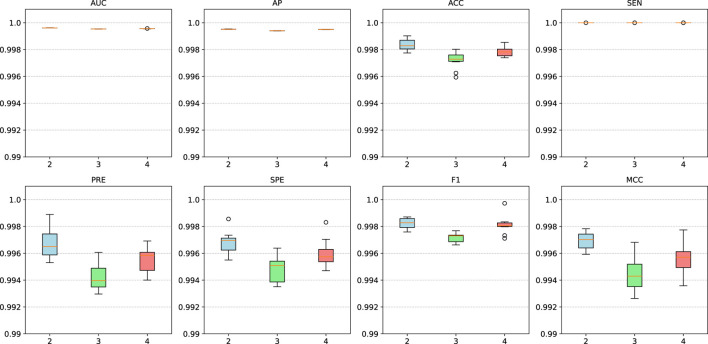
Model performance with different layers of GNN encoders.

We conducted additional comparative experiments to assess the impact of varying GNN encoder layers, with results shown in Figure 5. The findings indicate that when the number of GNN layers is set between 2 and 4, the model maintains stable performance without significant fluctuations. This demonstrates the model’s robustness to layer variations, suggesting that its performance remains largely unaffected by this parameter.

**FIGURE 6:**
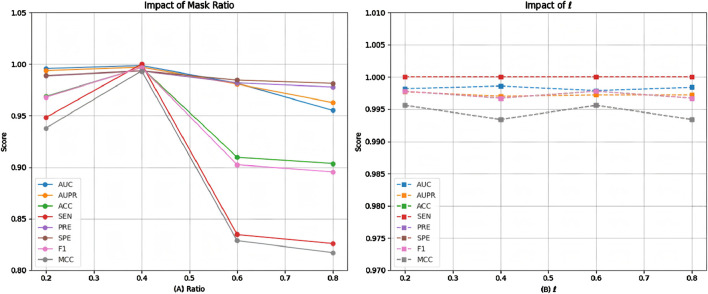
Model performance of at **(A)** different masking ratios and **(B)** weight parameter l .

The proposed model applies random edge sampling and masking in the metabolite-disease network based on the Bernoulli distribution, following a predetermined ratio. To assess the impact of different masking ratios on model performance, we conducted multiple comparative experiments, with the results presented in Figure 6A. The findings indicate that performance improves as the masking ratio increases from 0.2 to 0.4. However, beyond 0.4, performance begins to decline, with a sharper decrease observed between 0.4 and 0.6, followed by a more gradual decline from 0.6 to 0.8. This suggests that a masking ratio of 0.4 is optimal. A lower masking ratio may fail to effectively mitigate noise interference, whereas a higher ratio may result in critical topological information loss. Therefore, selecting an appropriate masking ratio is essential to balance noise reduction and information retention.

Since the proposed model employs a dual-decoder architecture during training, we conducted multiple comparative experiments to assess its performance stability under different neighborhood decoder weight settings. As shown in Figure 6B, the results indicate that when the neighborhood decoder weights range from 0.2 to 0.8, the model maintains stable performance with no significant fluctuations. This suggests that the model is robust to variations in this parameter, making it relatively straightforward to determine an appropriate weight for the neighborhood decoder.

### Case analysis

In this study, we performed in-depth case analyses on Maple Syrup Urine Disease (MSUD) and Cirrhosis, focusing on their associated metabolite components. MSUD is a hereditary amino acid metabolic disorder caused by a deficiency in branched-chain α-keto acid dehydrogenase (BCKD), leading to the accumulation of branched-chain amino acids (BCAAs) and resulting in neurological damage (Blackburn et al., 2017). Early and accurate diagnosis of metabolites plays a crucial role in treatment and dietary management, helping to control the disease effectively. To explore this, we first excluded all metabolites related to MSUD from the training set and trained the model. Subsequently, we introduced MSUD-related metabolites into the test set and used the trained model to predict them, sorting the metabolites by prediction scores and selecting the top 20. As shown in Table 3, 18 of the predicted metabolites were validated in the database. For example, Deng et al. achieved rapid and accurate MSUD diagnosis by measuring L-phenylalanine, L-valine, and L-leucine in newborns, using only small sample sizes (Deng and Deng, 2003). Although (S)-3-hydroxyisobutyric acid and heparan sulfate were not validated in the database, studies suggest that (S)-3-hydroxyisobutyric acid plays a key role in the metabolic pathway of L-valine (Gibson et al., 1993), indicating its potential as an early marker for diseases like MSUD.

**TABLE 3:** Top 20 predicted metabolites with potential associations with MSUD.

Metabolites	HMDB	Metabolites	HMDB
1-Methylhistidine	Confirmed	L-Valine	Confirmed
Betaine	Confirmed	Hippuric acid	Confirmed
Glycine	Confirmed	Ethanolamine	Confirmed
Taurine	Confirmed	3-Methyl-2-oxovaleric acid	Confirmed
Ketoleucine	Confirmed	2-Hydroxy-3-methylbutyric acid	Confirmed
L-Phenylalanine	Confirmed	alpha-Ketoisovaleric acid	Confirmed
L-Arginine	Confirmed	(S)-3-Hydroxyisobutyric acid	Unconfirmed
L-Alloisoleucine	Confirmed	Acetic acid	Confirmed
L-Leucine	Confirmed	Trimethylamine N-oxide	Confirmed
L-Glutamine	Confirmed	Heparan sulfate	Unconfirmed

Cirrhosis is a chronic liver disease often caused by viral hepatitis, excessive alcohol consumption, and unhealthy dietary habits, such as high-fat intake. Early symptoms are typically subtle, but as the disease progresses to the decompensated stage, severe complications like ascites, gastrointestinal bleeding, and liver cancer may arise. Early metabolite-based diagnosis is crucial for guiding treatment and dietary management to slow disease progression. This study excluded all metabolites associated with Cirrhosis from the training set before proceeding with model training. During testing, these metabolites were reintroduced into the test set, and the trained model was used to predict them. The predictions were ranked by score, and the top 20 metabolites were selected. As shown in Table 4, 19 of the predicted metabolites were validated in the database. For instance, Tamasawa et al. found that Cholesterol Sulfate (CS) levels significantly differed between patients with high cholesterol and those with Cirrhosis (Tamasawa et al., 1993), suggesting that CS could serve as an early diagnostic biomarker.

**TABLE 4:** Top 20 predicted metabolites with potential associations with Cirrhosis.

Metabolites	HMDB	Metabolites	HMDB
Deoxypyridinoline	Confirmed	L-Aspartic acid	Confirmed
Sulfolithocholylglycine	Confirmed	Taurocholic acid	Confirmed
Deoxycholic acid glycine conjugate	Confirmed	Glycochenodeoxycholate-3-sulfate	Confirmed
Cholesterol sulfate	Confirmed	Argininic acid	Confirmed
3,5-Diiodothyronine	Confirmed	Methanethiol	Confirmed
Fructosamine	Confirmed	L-Urobilinogen	Confirmed
2,3-Butanediol	Confirmed	2-Oxoarginine	Confirmed
L-Palmitoylcarnitine	Confirmed	D-Urobilin	Confirmed
Acetic acid	Unconfirmed	Elaidic carnitine	Confirmed
Cholic acid	Confirmed	Creatine	Confirmed

**FIGURE 7:**
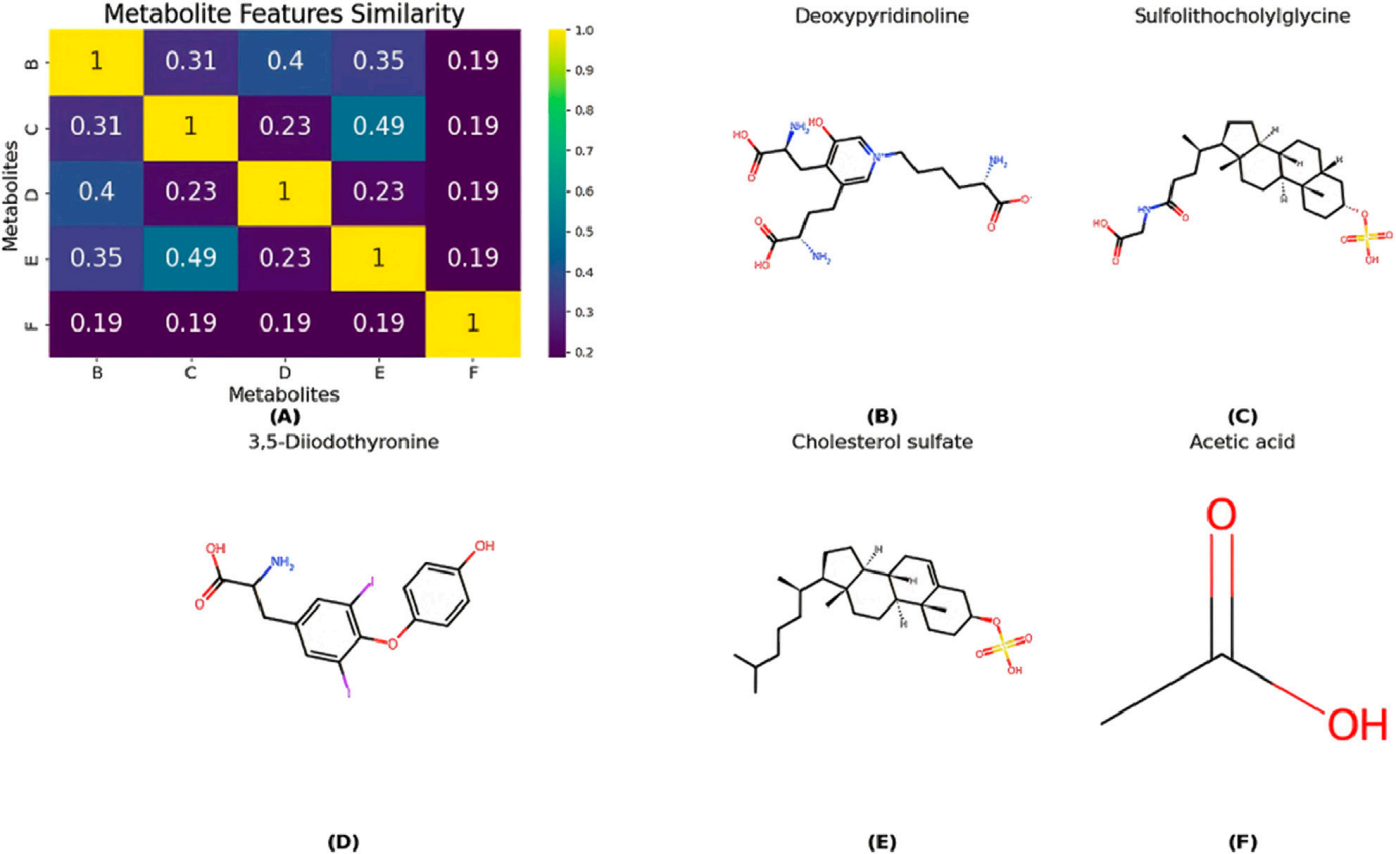
**(A)** The similarity between predicted metabolites and known Cirrhosis-related metabolites. And panels **(B–F)** correspond to the chemical structures of the following compounds: deoxypyridinoline, sulfolithocholylglycine, 3,5-diiodothyronine, cholesterol sulfate, and acetic acid.

Although Acetic acid has not been validated in the database, it is a metabolite of ethanol and plays a role in various metabolic pathways related to Cirrhosis. Thus, measuring acetic acid levels may help infer Cirrhosis or other metabolic diseases. Furthermore, Figure 7 illustrates the similarity between predicted metabolites and known Cirrhosis-related metabolites. Notably, Deoxypyridinoline, Sulfolithocholylglycine, 3,5-Diiodothyronine, and Cholesterol sulfate exhibit high similarity, whereas Acetic acid shows lower similarity.

We further investigated disease associations for selected metabolites and focused on two key metabolites: deoxyguanosine and dihydrobiopterin. Deoxyguanosine, a DNA nucleoside composed of guanine and deoxyribose, serves as a biosynthetic precursor for deoxyguanosine triphosphate (dGTP), an essential DNA synthesis substrate (Greenberg, 2004). Dihydrobiopterin (BH_2_), a crucial biopterin cycle intermediate, regulates neurotransmitter synthesis and vascular function (Fismen et al., 2012). The BH_2_/BH_4_ balance represents a promising therapeutic target for neurological and cardiovascular disorders. We first excluded all known MDAs for these metabolites from the training set. The model then predicted potential disease associations, with the top 10 predictions for each metabolite shown in Table 5. Notably, 7 deoxyguanosine-related and 8 dihydrobiopterin-related disease predictions were experimentally validated. These case studies further validate the potential of the proposed model in uncovering MDAs, offering valuable insights for natural medicine development.

**TABLE 5:** Top 10 predicted diseases with potential associations with metabolites Deoxyguanosine and Dihydrobiopterin, respectively.

Diseases (deoxyguanosine)	HMDB	Diseases (dihydrobiopterin)	HMDB
Lewy body disease	Confirmed	Irritable bowel syndrome	Confirmed
Canavan disease	Confirmed	Crohn’s disease	Confirmed
Alzheimer’s disease	Confirmed	Eosinophilic esophagitis	Confirmed
Frontotemporal dementia	Confirmed	Rheumatoid arthritis	Confirmed
Cystinosis	Unconfirmed	Autism	Confirmed
Ulcerative colitis	Confirmed	Colorectal cancer	Confirmed
Colorectal cancer	Confirmed	Degenerative disc disease	Unconfirmed
Galactosemia	Unconfirmed	AIDS	Confirmed
Crohn’s disease	Confirmed	Celiac disease	Confirmed
Osteoporosis	Unconfirmed	Rhinitis	Unconfirmed

## Conclusion

Diabetes and other metabolic diseases pose significant threats to human health, with their complex pathological mechanisms presenting challenges for combination drug therapy. Natural medicines, which often contain multiple active components and have fewer side effects, offer a promising treatment approach. Since metabolic disorders are closely linked to disease pathogenesis, analyzing metabolic product levels not only aids in diagnosis but also enhances our understanding of the metabolic regulation mechanisms underlying natural medicines. This knowledge can inform targeted strategies for preventing and treating metabolic diseases. In this study, we propose a novel method based on GAE technology to elucidate the pathological mechanisms of metabolic diseases through metabolite analysis. By leveraging known MDAs, we apply NMF to extract initial features, which are then integrated into a GAE model to systematically capture potential disease mechanisms.

Our experimental results demonstrate effective identification of disease-related patterns and complex metabolic interactions. Case studies further validate the model’s capability to elucidate pathological mechanisms in diabetes and other metabolic disorders. Nevertheless, our model has several limitations: (1) Limited generalizability of initial feature representations; (2) Dependence solely on topological information without multi-source data integration. To address these limitations, we propose the following future work: (i) Employing large language models to learn general metabolite/disease knowledge for robust feature extraction; (ii) Developing multimodal fusion approaches incorporating SMILES sequences, 2D/3D structures, and clinical data for enhanced representations. These advancements will deepen our understanding of disease mechanisms and facilitate natural medicine discovery, potentially leading to improved therapeutic strategies.

## Data Availability

The original contributions presented in the study are included in the article/supplementary material, further inquiries can be directed to the corresponding authors.
